# Association of Cardiac Rehabilitation With Survival Among US Veterans

**DOI:** 10.1001/jamanetworkopen.2020.1396

**Published:** 2020-03-20

**Authors:** Nirupama Krishnamurthi, David W. Schopfer, Hui Shen, Mary A. Whooley

**Affiliations:** 1 San Francisco Veterans Affairs Medical Center, San Francisco, California; 2 Icahn School of Medicine at Mount Sinai St Luke’s and Mount Sinai West, New York, New York; 3 Department of Medicine, University of California, San Francisco; 4 Department of Epidemiology and Biostatistics, University of California, San Francisco

## Abstract

**Importance:**

Participation in cardiac rehabilitation (CR) programs at Veterans Affairs (VA) facilities is low. Most veterans receive CR through purchased care at non-VA programs. However, limited literature exists on the comparison of outcomes between VA and non-VA CR programs.

**Objective:**

To compare 1-year mortality and 1-year readmission rates for myocardial infarction or coronary revascularization between VA vs non-VA CR participants.

**Design, Setting, and Participants:**

This cohort study included 7320 patients hospitalized for myocardial infarction or coronary revascularization at the VA between 2010 and 2014 who did not die within 30 days of discharge and who participated in 2 or more CR sessions after discharge. The study excluded individuals hospitalized for ischemic heart disease after December 2014 when the VA Choice Act changed referral criteria for non-VA care. Data analysis was performed from November 2019 to January 2020.

**Exposures:**

Participation in 2 or more CR sessions within 12 months of discharge at a VA or non-VA facility.

**Main Outcomes and Measures:**

The 1-year all-cause mortality and 1-year readmission rates for myocardial infarction or coronary revascularization from date of discharge were compared between VA vs non-VA CR participants using Cox proportional hazards models with inverse probability treatment weighting.

**Results:**

The 7320 veterans with ischemic heart disease who participated in CR programs had a mean (SD) age of 65.13 (8.17) years and were predominantly white (6005 patients [82.0%]), non-Hispanic (6642 patients [91.0%]), and male (7191 patients [98.2%]). Among these 7320 veterans, 2921 (39.9%) attended a VA facility, and 4399 (60.1%) attended a non-VA CR facility. Black and Hispanic veterans were more likely to attend CR programs at VA facilities (509 patients [17.4%] and 378 patients [12.9%], respectively), whereas white veterans were more likely to attend CR programs at non-VA facilities (3759 patients [85.5%]). After inverse probability treatment weighting, rates of 1-year mortality were 1.7% among VA CR participants vs 1.3% among non-VA CR participants (hazard ratio, 1.32; 95% CI, 0.90-1.94; *P* = .15). Rates of readmission for myocardial infarction or revascularization during the 12 months after discharge were 4.9% among VA CR participants vs 4.4% among non-VA CR participants (hazard ratio, 1.06; 95% CI, 0.83-1.35; *P* = .62).

**Conclusions and Relevance:**

These findings suggest that rates of 1-year mortality and 1-year readmission for myocardial infarction or revascularization did not differ for participants in VA vs non-VA cardiac rehabilitation programs. Eligible patients with ischemic heart disease should participate in CR programs regardless of where they are provided.

## Key Points

**Question:**

Among veterans with ischemic heart disease, do the morbidity and mortality benefits associated with participation in a cardiac rehabilitation program differ between Veterans Affairs (VA) (delivered care) vs non-VA (purchased care) settings?

**Findings:**

In this cohort study of 7320 US veterans, rates of 1-year mortality and 1-year readmission for myocardial infarction or revascularization did not differ for participants in VA vs non-VA cardiac rehabilitation programs.

**Meaning:**

These findings suggest that participation in VA vs non-VA cardiac rehabilitation programs is associated with similar 1-year benefits for morbidity and mortality.

## Teaser

This cohort study examines 1-year mortality and 1-year readmission rates for myocardial infarction or coronary revascularization for US veterans participating in Veterans Health Administration vs non–Veterans Health Administration cardiac rehabilitation programs.

## Introduction

Cardiac rehabilitation (CR) is a multidisciplinary secondary prevention program aimed at reducing cardiovascular risk in patients with preexisting heart disease. Exercise-based CR has been shown to reduce cardiovascular-associated mortality and hospitalizations and improve quality of life in patients with coronary heart disease.^1,2^ Referral to CR is a class I recommendation from the American Heart Association and the American College of Cardiology for patients with recent myocardial infarction (MI), percutaneous coronary intervention (PCI), coronary artery bypass grafting (CABG), chronic stable angina, or heart failure.^3,4,5^

Historically, participation in CR programs in the US Department of Veterans Affairs (VA) has been reported to be low among patients with ischemic heart disease, with substantial geographic variation across the country.^6,7,8^ The VA is adopting innovative means to improve CR participation; however, because not all VA facilities offer CR and a large proportion of veterans live far from a VA facility, referral to a CR program outside the VA (non-VA CR) is a common practice. Evaluation of CR outcomes in the VA (including non-VA referrals) is important to inform policy decisions and clinical care, particularly in the setting of recent changes in VA policy resulting in a potential increase in the use of community care referrals for health care in the VA.

Limited literature currently exists on the comparison of outcomes between VA-delivered and non-VA (purchased community care) CR programs. Therefore, we conducted a cohort study using the national VA electronic health record to compare 1-year all-cause mortality and 1-year readmission rates for non-fatal MI, PCI, and/or CABG among veterans attending VA and non-VA CR programs.

## Methods

 This study was approved by the San Francisco Veterans Health Administration and University of California, San Francisco institutional review boards. The requirement for informed consent was waived because the research involved no more than minimal risk to the participants, the waiver did not adversely affect the rights or welfare of the participants, and the research could not practicably be performed without the waiver. This study follows the Strengthening the Reporting of Observational Studies in Epidemiology (STROBE) reporting guideline for cohort studies.

We extracted electronic health record data from the national VA Corporate Data Warehouse for this study. Patients hospitalized for MI, PCI, or CABG at a VA hospital between 2010 and 2014 were identified using the following *International Classification of Diseases, Ninth Revision* (*ICD-9*), *Current Procedural Terminology* (*CPT*), and *Healthcare Common Procedure Coding System* (*HCPCS*) codes: MI (*ICD-9 *code 410.x), PCI (*ICD-9* codes 00.66, 17.55, and 36.0x; *CPT* codes 92973, 92974, 92980-92982, 92984, 92995, and 92996; *HCPCS* codes G0290 and G0291), and CABG (*ICD-9* codes 36.10-36.16, 36.19, and 36.20; *CPT* codes 33510-33514, 33516-33519, 33521-33523, 33530, 33533-33536, 33572, 35600, and 93564; *HCPCS* codes S2205-2209). To ensure that all patients in the study had similar access to CR, we did not include individuals hospitalized for ischemic heart disease after December 2014, when the VA Choice Act was implemented and substantially changed the approval criteria for veterans to seek purchased care at non-VA hospitals.

Patients who died within 30 days of discharge were excluded from the analysis. Among the remaining patients, we identified those who participated in 2 or more CR sessions within 12 months of discharge using *CPT* codes 93797, 93798, S9472, S9473, G0422, and G0423 and classified them as CR participants. Because visits and intake sessions without any exercise are often recorded using the same codes as those with exercise training, we required 2 or more encounters associated with the CR codes to qualify as participation to ensure at least 1 session of exercise training and/or behavioral education. Patients hospitalized for MI, PCI, or CABG between 2010 and 2014 who had only 1 or no CR sessions within 12 months of discharge were excluded from the analysis. Any CR encounters obtained from the VA outpatient tables were classified as VA CR, whereas those obtained from Fee Basis files (containing information about health care visits outside the VA that were paid for by the VA) were categorized as non-VA CR. The number of CR sessions was calculated from the first session up to a period of 6 months after the first session. For patients who had multiple events (ie, ≥2 hospitalizations for MI, PCI, or CABG) between 2010 and 2014, we selected the first episode of CR participation.

Information on patient demographic characteristics, geographical location, and comorbid conditions (defined as conditions coded in 2 outpatient and/or 1 inpatient encounter in the 12 months before hospitalization) was obtained from the Corporate Data Warehouse files. Mortality and readmissions for major adverse cardiovascular events, including hospitalization for acute MI or coronary revascularization in VA or non-VA settings, were assessed during the 12 months after discharge. Patients who died before starting CR were excluded. Readmissions were counted only if they occurred after at least 2 CR sessions.

### Statistical Analysis

Baseline characteristics of participants were compared using χ^2^ tests for categorical variables and 2-sided *t* tests for continuous variables, with *P* < .05 considered statistically significant. Stabilized propensity weights were generated for VA CR vs non-VA CR participants using data on patient demographic characteristics, regional distribution, indication for CR, and comorbid conditions. We used Cox proportional hazards models with inverse probability treatment weighting to compare mortality and readmissions for VA CR participants with those for non-VA CR participants. Sensitivity analysis was performed by stratifying participants into 2 groups according to time to first CR session from discharge (0-6 months vs >6 months) and then comparing mortality and readmission outcomes between VA and non-VA CR participants within the 2 strata. All statistical analyses were performed using SAS Enterprise Guide statistical software version 7.15 HF3 (SAS Institute) and Stata statistical software version 15.1 (StataCorp). Data analyses were performed from November 2019 to January 2020.

## Results

Between 2010 and 2014, 84 489 patients were hospitalized at a VA facility in the US for MI, PCI, or CABG. Among these eligible patients, 7320 (8.7%) participated in 2 or more CR sessions. Patients who participated in CR had a mean (SD) age of 65.13 (8.17) years and were predominantly white (6005 patients [82.0%]), non-Hispanic (6642 patients [91.0%]), and male (7191 patients [98.2%]) (Table 1). Of the 7320 CR participants, 2921 (39.9%) attended CR programs at a VA facility, and the remainder 4399 (60.1%) attended a non-VA CR facility. Black and Hispanic veterans were more likely to attend CR programs at VA facilities (509 patients [17.4%] and 378 patients [12.9%], respectively), whereas white veterans were more likely to attend non-VA facilities (3759 patients [85.5%]). Between 2010 and 2014, the number of non-VA CR participants steadily increased (from 766 veterans in 2010 to 1032 veterans in 2014), whereas the number of VA CR participants remained stable (596 veterans in 2010 and 614 veterans in 2014). The 2 groups were similar in terms of the distribution of comorbid conditions; however, non-VA CR participants had higher rates of comorbid hypertension (3212 patients [73.0%] vs 1958 patients [67.0%]) and dyslipidemia (3122 patients [71.0%] vs 1975 patients [67.6%]), whereas VA CR participants had more chronic kidney disease (371 patients [12.7%] vs 459 patients [10.4%]).

**Table 1.** Characteristics of Veterans Attending CR Programs at VA vs Non-VA Facilities

Characteristic	CR program participants, No. (%)			*P* value
	Total (N = 7320)	VA (n = 2921)	Non-VA (n = 4399)	
Age, mean (SD), y	65.13 (8.17)	65.26 (8.39)	65.04 (8.02)	.26
Male	7191 (98.2)	2866 (98.1)	4325 (98.3)	.52
Race^a^				
White	6005 (82.0)	2246 (76.9)	3759 (85.5)	<.001
Black	868 (11.9)	509 (17.4)	359 (8.2)	
Other	319 (4.4)	129 (4.4)	190 (4.3)	
Ethnicity, Hispanic or Latino^b^	508 (6.9)	378 (12.9)	130 (3.0)	<.001
Marital status, married^c^	4092 (55.9)	1459 (49.9)	2633 (59.9)	<.001
Year of discharge				
2010	1362 (18.6)	596 (20.4)	766 (17.4)	.001
2011	1422 (19.4)	587 (20.1)	835 (19.0)	
2012	1387 (18.9)	560 (19.2)	827 (18.8)	
2013	1503 (20.5)	564 (19.3)	939 (21.3)	
2014	1646 (22.5)	614 (21.0)	1032 (23.5)	
VA region				
Midwest	2703 (36.9)	805 (27.6)	1898 (43.1)	<.001
Southeast	1730 (23.6)	786 (26.9)	944 (21.5)	
North Atlantic	1252 (17.1)	420 (14.4)	832 (18.9)	
Continental	975 (13.3)	637 (21.8)	338 (7.7)	
Pacific	660 (9.0)	273 (9.3)	387 (8.8)	
Indication for CR				
Acute myocardial infarction	2435 (33.3)	981 (33.6)	1454 (33.1)	.04
Coronary artery bypass grafting	2613 (35.7)	1080 (37.0)	1533 (34.8)	
Percutaneous coronary intervention	2272 (31.0)	860 (29.4)	1412 (32.1)	
Comorbid conditions				
Hypertension	5170 (70.6)	1958 (67.0)	3212 (73.0)	<.001
Dyslipidemia	5097 (69.6)	1975 (67.6)	3122 (71.0)	.002
Diabetes	3060 (41.8)	1186 (40.6)	1874 (42.6)	.09
Heart failure	995 (13.6)	435 (14.9)	560 (12.7)	.01
Stroke	298 (4.1)	140 (4.8)	158 (3.6)	.01
Peripheral vascular disease	808 (11.0)	297 (10.2)	511 (11.6)	.05
Chronic obstructive pulmonary disease	1003 (13.7)	382 (13.1)	621 (14.1)	.21
Chronic kidney disease	830 (11.3)	371 (12.7)	459 (10.4)	.002
Valvular heart disease	908 (12.4)	351 (12.0)	557 (12.7)	.41
Arrhythmias	1259 (17.2)	474 (16.2)	785 (17.8)	.07
Cancer	810 (11.1)	358 (12.3)	452 (10.3)	.01
Dementia	32 (0.4)	15 (0.5)	17 (0.4)	.42
Anemia	837 (11.4)	360 (12.3)	477 (10.8)	.05
Depression	1466 (20.0)	607 (20.8)	859 (19.5)	.19
Posttraumatic stress disorder	925 (12.6)	348 (11.9)	577 (13.1)	.13
Alcohol abuse or dependence	419 (5.7)	186 (6.4)	233 (5.3)	.05
Smoking	1968 (26.9)	750 (25.7)	1218 (27.7)	.06
Time from discharge to first CR session, median (interquartile range), d	61 (37-105)	63 (40-106)	60 (35-105)	.27
1-y Mortality	122 (1.7)	56 (1.9)	66 (1.5)	.17
1-y Readmissions total	346 (4.7)	148 (5.1)	198 (4.5)	.26
Myocardial infarction	112 (1.5)	41 (1.4)	71 (1.6)	.47
Coronary bypass grafting	19 (0.3)	3 (0.1)	16 (0.4)	.03
Percutaneous coronary intervention	215 (2.9)	104 (3.6)	111 (2.5)	.01

Abbreviations: CR, cardiac rehabilitation; VA, Veterans Affairs.

^a^ Data on race were missing for 128 participants (37 VA participants and 91 non-VA participants).

^b^ Data on ethnicity were missing for 170 participants (66 VA participants and 104 non-VA participants).

^c^ Data on marital status were missing for 1 VA participant.

Time from index event to CR enrollment was similar in VA vs non-VA programs (63 vs 60 days). The unadjusted 1-year mortality rate was similar between VA and non-VA CR participants (1.9% vs 1.5%). Similarly, the unadjusted 1-year overall readmission rates for MI, PCI, or CABG did not differ between VA and non-VA CR participants (5.1% vs 4.5%). However, the unadjusted readmission rate for CABG was lower (0.1% vs 0.4%), and the readmission rate for PCI was higher (3.6% vs 2.5%), among VA vs non-VA participants (Table 1).

**Figure 1.**
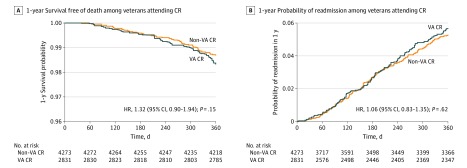
Probabilities of 1-Year Survival and Readmission A, One-year survival free of death among veterans attending cardiac rehabilitation (CR) at Veterans Affairs (VA) vs non-VA facilities from Cox proportional hazards model with inverse probability of treatment weighting. B, One-year probability of readmission for acute myocardial infarction, percutaneous coronary intervention, or coronary artery bypass grafting in veterans attending CR at VA vs non-VA facilities from Cox proportional hazards model with inverse probability of treatment weighting. Two hundred sixteen patients were excluded for missing demographic data. HR indicates hazard ratio.

On applying inverse probability treatment weighting, both groups were similar in their weighted distribution of demographic characteristics and comorbid conditions (Table 2). Rates of 1-year mortality were 1.7% among VA vs 1.3% among non-VA CR participants (hazard ratio [HR], 1.32; 95% CI, 0.90-1.94; *P* = .15) (Figure 1A and Table 3). Rates of readmission for MI or revascularization during the 12 months after discharge were 4.9% among VA participants vs 4.4% among non-VA CR participants (HR, 1.06; 95% CI, 0.83-1.35; *P* = .62) (Figure 1B and Table 3). Separate Cox proportional hazard models estimating the hazard of readmission for MI and CABG did not show any statistically significant difference between the 2 groups; however, the hazard of 1-year readmission for PCI was higher among VA CR participants than non-VA CR participants (HR, 1.45; 95% CI, 1.08-1.94; *P* = .01).

**Table 2.** Weighted Distribution of Participant Characteristics by CR Delivery Site

Characteristic	CR program participants, %		*P* value
	VA	Non-VA	
Age, mean (SD), y	64.94 (8.14)	64.96 (8.22)	.90
Male	98.1	98.2	.74
Race			
White	83.8	84.0	.97
Black	12.1	12.0	
Other	4.1	4.0	
Ethnicity, Hispanic or Latino	6.9	8.5	.07
Marital status, married	55.6	55.3	.80
Year of discharge			
2010	18.0	17.8	.97
2011	18.8	18.2	
2012	18.7	18.9	
2013	20.9	21.0	
2014	23.6	24.1	
VA region			
Midwest	37.2	36.4	.26
Southeast	23.5	22.0	
North Atlantic	16.5	16.8	
Continental	13.4	15.4	
Pacific	9.4	9.4	
Indication for CR			
Acute myocardial infarction	33.7	34.0	.93
Coronary artery bypass grafting	35.3	34.8	
Percutaneous coronary intervention	30.9	31.1	
Comorbid conditions			
Hypertension	70.4	70.1	.83
Dyslipidemia	68.9	68.9	.96
Diabetes	41.5	41.5	.98
Heart failure	13.4	13.3	.92
Stroke	4.1	4.3	.70
Peripheral vascular disease	11.0	10.9	.89
Chronic obstructive pulmonary disease	13.7	13.9	.88
Chronic kidney disease	11.4	11.1	.75
Valvular heart disease	12.4	12.7	.73
Arrhythmias	17.4	17.2	.85
Cancer	11.1	10.8	.79
Dementia	0.4	0.4	.90
Anemia	11.6	11.6	.96
Depression	20.2	20.1	.92
Posttraumatic stress disorder	12.5	13.0	.54
Alcohol abuse or dependence	6.1	6.4	.65
Smoking	26.9	27.1	.85
1-y Mortality	1.7	1.3	.15
1-y Readmission	4.9	4.4	.35

Abbreviations: CR, cardiac rehabilitation; VA, Veterans Affairs.

**Table 3.** Mortality and Readmissions at 1 Year Among All Patients Who Underwent CR at VA vs Non-VA Facilities and Among Patients Who Started CR Within 6 Months of Discharge

Characteristic	All patients		Patients who started CR within 6 mo	
	HR (95% CI)	*P* value	HR (95% CI)	*P* value
1-y Mortality	1.32 (0.90-1.94)	.15	1.33 (0.90-1.97)	.15
1-y Readmission total	1.06 (0.83-1.35)	.62	1.06 (0.82-1.35)	.66
Myocardial infarction	0.71 (0.45-1.12)	.14	0.71 (0.44-1.14)	.16
Coronary bypass grafting	0.28 (0.07-1.10)	.07	0.29 (0.07-1.14)	.07
Percutaneous coronary intervention	1.45 (1.08-1.94)	.01	1.46 (1.08-1.96)	.01

Abbreviations: CR, cardiac rehabilitation; HR, hazard ratio; VA, Veterans Affairs.

**Figure 2.**
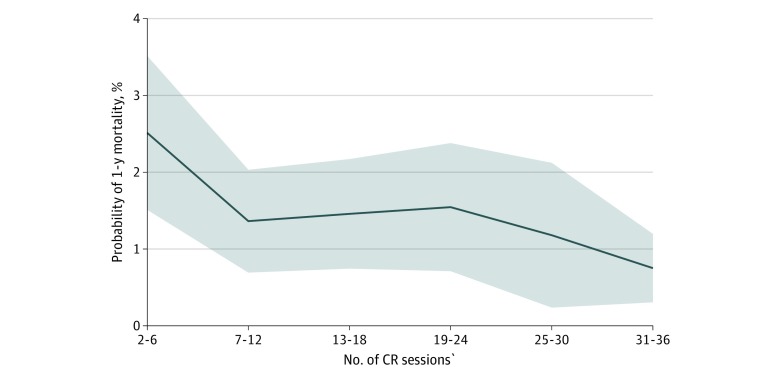
Probability of Mortality at 1 Year by Number of Cardiac Rehabilitation (CR) Sessions Adjusted marginal probability of 1-year mortality (line) with 95% CIs (shaded areas), by number of CR sessions attended.

On stratifying patients by time from discharge to first CR session, we found that 6561 patients (89.6%) started CR within 6 months of discharge and the remaining 759 patients (10.3%) started CR after 6 months. We found no differences in 1-year mortality (HR, 1.33; 95% CI, 0.90-1.97; *P* = .15) or 1-year overall readmission (HR, 1.06; 95% CI, 0.82-1.35; *P* = .66) rates for MI or revascularization between VA and non-VA CR participants in either strata (Table 3). Figure 2 shows the probability of mortality at 1 year according to the number of CR sessions.

## Discussion

Among patients who were hospitalized at a VA facility between 2010 and 2014 for MI, PCI, or CABG, only 8.7% participated in CR, with 39.9% of participants attending VA-delivered CR programs and 60.1% attending non-VA (purchased care) CR programs. Black and Hispanic veterans were more likely to attend VA CR, whereas white veterans were more likely to attend non-VA CR, suggesting that VA-delivered care may address some of the racial and ethnic disparities seen in prior studies. There were no statistically significant differences in rates of 1-year mortality or 1-year overall readmissions for major adverse cardiovascular events between those who attended CR in VA vs non-VA settings. These findings highlight the need to redouble efforts to improve participation in CR, regardless of where it is provided, among eligible patients with ischemic heart disease.

Prior studies^1,2,9,10,11,12,13,14,15^ have established the association of CR with reduced cardiovascular mortality. Several studies^16,17,18,19,20,21,22,23^ have compared the quality of care between VA and non-VA settings; however, to our knowledge, this study is the first to compare outcomes of CR between VA and non-VA facilities. A systematic review comparing the quality of VA and non-VA care found that mortality outcomes were comparable between the 2 settings or favored VA care, depending on the condition studied.^21^ A study^22^ from 2007 found higher mortality rates in VA facilities among patients who underwent revascularization procedures. However, 2 newer studies^20,24^ looking at 30-day mortality outcomes found that compared with non-VA care, VA care was associated with better survival among patients hospitalized for MI or PCI. One study^20^ found higher readmission rates for MI in the VA compared with non-VA hospitals.

Given the heterogeneity in outcomes between VA and non-VA care, it is reassuring to find that participation in CR programs in either setting is associated with similar all-cause mortality and overall readmission rates for MI or revascularization. This is particularly relevant in light of the fact that most VA medical centers do not have on-site CR programs, necessitating referral to non-VA programs.^7,8^ In addition, a qualitative study^25^ looking at barriers to CR participation in the VA found that the most common reason for refusal was patient transportation issues. Because there is discussion around expanding community care in the VA alongside the impetus to increase CR participation, our research can help inform both practitioner and patient decisions around referral to CR.

Our results included a higher probability of 1-year readmission for PCI among VA CR participants than non-VA CR participants. However, our analysis only captured readmissions at VA hospitals or non-VA hospitals that were reimbursed by the VA. Veterans who attended non-VA CR may have been more likely than those who attended VA CR programs to have rehospitalizations covered through private insurance or Medicare, in which case we could have undercounted readmissions in the non-VA care group. Given that the mortality rates and 1-year MI readmission rates were similar between the 2 groups, the finding that VA CR participants were readmitted more often for PCI (at VA facilities or those reimbursed by the VA) should not be viewed as an indicator of a worse health outcome.

### Limitations

There are several limitations to our study. Because we used electronic health records to obtain nationwide data for this study, we were unable to account for intangible factors, such as patient motivation and psychological factors that play an important role in CR participation and completion. We were also unable to account for data such as numbers of vessels revascularized, ejection fraction, or medication adherence, which are important measures of disease severity. However, we adjusted for CR indication and traditional risk factors for cardiovascular disease, which would, in part, reflect disease severity. Furthermore, CR is available at fewer than one-half of VA facilities, and we did not have information on which facilities offered formal CR programs. Thus, we were unable to include program availability or distance to VA CR programs in our propensity model.

## Conclusions

This study found that VA and non-VA CR programs were associated with similar 1-year mortality and 1-year overall readmission rates for MI, PCI, or CABG. We hope that these findings encourage greater referral of all eligible patients to CR, either at the VA or outside the VA, and promote adherence to CR programs. In addition, we hope that this information helps patients make an informed decision about participation in and completion of CR programs.
